# Alanine-Serine-Cysteine Transporter 2 Inhibition Suppresses Prostate Cancer Cell Growth In Vitro

**DOI:** 10.3390/jcm11185466

**Published:** 2022-09-16

**Authors:** Masanobu Saruta, Kiyoshi Takahara, Atsuhiko Yoshizawa, Atsuko Niimi, Toshiyuki Takeuchi, Takuhisa Nukaya, Masashi Takenaka, Kenji Zennami, Manabu Ichino, Hitomi Sasaki, Mamoru Kusaka, Motoshi Suzuki, Makoto Sumitomo, Ryoichi Shiroki

**Affiliations:** 1 Department of Urology, Fujita-Health University School of Medicine, Nagoya 470-1192, Japan; 2 Department of Molecular Oncology, Fujita Health University School of Medicine, Nagoya 470-1192, Japan; 3 Department of Urology, Okazaki Medical Center, Fujita Health University, Okazaki 444-0827, Japan

**Keywords:** ASCT2, ARV7, AR, enzalutamide, prostate cancer

## Abstract

Alanine-serine-cysteine transporter 2 (ASCT2) has been associated with increased levels of metabolism in various malignant tumors. However, its biological significance in the proliferation of prostate cancer (PCa) cells remains under investigation. We used the cBioPortal database to assess the effect of ASCT2 expression on the oncological outcomes of 108 PCa patients. To evaluate the function of ASCT2 in castration-sensitive PCa (CSPC) and castration-resistant PCa (CRPC), LNCaP cells and the ARV7-positive PCa cell line, 22Rv1, were assessed using cell proliferation assays and Western blot analyses. The ASCT2 expression level was associated with biochemical recurrence-free survival after prostatectomy in patients with a Gleason score ≥ 7. In vitro experiments indicated that the growth of LNCaP cells after combination therapy of ASCT2 siRNA and enzalutamide treatment was significantly reduced, compared to that following treatment with enzalutamide alone or ASCT2 siRNA transfection alone (*p* < 0.01, 0.01, respectively). After ASCT2 inhibition by siRNA transfection, the growth of 22Rv1 cells was significantly suppressed as compared with negative control siRNA via downregulation of ARV7 both in fetal bovine serum and androgen-deprivation conditions (*p* < 0.01, 0.01, respectively). We demonstrated that ASCT2 inhibition significantly reduced the proliferation rates of both CSPC and CRPC cells in vitro.

## 1. Introduction

Suppression of androgen receptor (AR) signaling through androgen deprivation therapy (ADT) remains the primary treatment for metastatic prostate cancer (mPCa). Although most patients with metastatic castration-sensitive prostate cancer (mCSPC) initially respond to ADT, the majority progress to metastatic castration-resistant prostate cancer (mCRPC), which is refractory to ADT within one year [1,2,3].

Most castration-resistant prostate cancers (CRPCs) rely on AR signaling due to several adaptive tumor responses that facilitate ligand availability and AR activation, foster the emergence of ligand-independent forms of AR activation, or have acquired broader ligand sensitivity [4]. Considering these findings, the clinical researchers have been developed, resulting in the approval of potent AR-targeted therapies for patients with mCRPC [5]. However, resistance to AR-targeted therapies is often acquired, and most mCRPCs still depend on AR activation. The biological behavior of mCRPC through the AR axis cannot yet be accurately predicted by estimating the increased or diminished expression level of any single gene or small number of genes.

Cancer cells are metabolically reprogrammed to stimulate proliferation [6,7]. Several reprogramming activations have been recognized as hallmarks of cancer. Recent studies have shown that cancer cells metabolize glutamine to fulfill their metabolic needs [8,9]. Moreover, recent reports have highlighted the importance of several other major metabolic pathways such as the tricarboxylic acid (TCA) cycle and glutamine metabolism in many malignancies [10,11]. Understanding how to interfere with cancer cell metabolic reprogramming mechanisms will result in the development of new strategies for the therapy of malignancies.

Alanine-serine-cysteine transporter 2 (ASCT2) is a Na+-dependent neutral amino acid transporter involved in the cellular uptake of neutral amino acids such as glutamine, and is the primary transporter of glutamine in cancer cells [12]. Previous studies have shown that high-expression of ASCT2 is observed in various tumors, including breast, melanoma, colorectal, pancreatic, tongue, and lung cancers [9,13,14,15,16,17]. Moreover, Teixeira et al. concluded that ASCT2 inhibition and the combination of ASCT2 inhibitors with other anti-tumor therapies might be promising antineoplastic strategies [18]. Several investigators have emphasized the role of glutamine transporters in prostate cancer (PCa) [19,20,21,22,23,24]. Saarinen et al. demonstrated that both high-and low-Gleason grade tumors expressed ASCT2 in patients with CSPC and histologically confirmed PCa who underwent PET/CT before prostatectomy [24]. On the other hand, Chu et al. showed that the expression of ASCT2 in radical prostatectomy specimens was associated with better outcomes, by measuring ASCT2 using two different radiotracers in prostate PET scans [23]. Accordingly, the relationship between the expression and biological function of ASCT2 in PCa is still under investigation.

Herein, we examined the expression level of ASCT2 and its effect on the AR axis in PCa to better understand the mechanism of action of ASCT2 on castration-sensitive prostate cancer (CSPC) and CRPC progression. We also investigated the relationship between AR, ARV7, and ASCT2 in CSPC and CRPC cell lines using chemically synthesized ASCT2 siRNA and enzalutamide.

## 2. Materials and Methods

### 2.1. Cell Lines

The human PCa cell lines LNCaP and 22Rv1 were purchased from American Type Culture Collection (ATCC, Manassas, VA, USA). Cells were maintained in RPMI 1640 medium supplemented with 10% fetal bovine serum (FBS) (Life Technologies, Burlington, ON, Canada), charcoal/dextran-treated FBS (CSS) (Thermo Fisher Scientific, Waltham, MA, USA), and 1% penicillin/streptomycin (Life Technologies) at 37 °C in a humidified atmosphere containing 5% CO_2_.

### 2.2. siRNA Transfection in LNCaP or 22Rv1 Cells

LNCaP and 22Rv1 cells were grown overnight in RPMI 1640 medium supplemented with 10% FBS or CSS without antibiotics (day 0). Chemically synthesized ASCT2 siRNA or negative control siRNA (Sigma-Aldrich, St. Louis, MO, USA) diluted in Opti-MEM I with Lipofectamine RNAiMAX (Invitrogen, Carlsbad, CA, USA) or Lipofectamine alone was added to each well and incubated for four hours (day 1). The siRNA ASCT2 targeting sequences (5′-3′) were GUCAGCAGCCUUUCGCUCA (sense), and UGAGCGAAAGGCUGCUGAC (antisense). The medium was then changed to RPMI 1640 supplemented with 10% FBS or CSS in order to perform cell assays or Western blotting analyses.

### 2.3. Cell Growth Assay

To monitor the growth of ASCT2 siRNA-transfected cells, LNCaP and 22Rv1 cells (5 × 10^4^ cells per well) were seeded into 6-well plates in RRPMI 1640 medium supplemented with 10% FBS or CSS without antibiotics overnight (day 0). Transfection was performed using 1, 10, or 50 nM ASCT2 siRNA, negative control siRNA, or Lipofectamine alone (day 1). The cells were cultured for four days at 37 °C in a humidified atmosphere containing 5% CO_2_, and cell proliferation was checked on day 4. Cell proliferation was evaluated by using the cell counting kit-8 (Dojindo Laboratories, Kumamoto, Japan), according to the manufacturer’s instructions. The absorbance was measured using a spectrophotometer and the level was 450.

To monitor the growth in cells treated with enzalutamide (Selleck Chemicals, Houston, TX, USA), LNCaP cells (5 × 10^4^ cells per well) were seeded into 6-well plates in RPMI 1640 medium supplemented with 10% FBS containing 1% penicillin/streptomycin overnight (day 0). On day 1, 20 μM enzalutamide was added to each plate, and cell proliferation was evaluated on day 3.

In order to monitor the growth of cells after the combination therapy of ASCT2 siRNA and enzalutamide, LNCaP cells (5 × 10^4^ cells per well) were seeded into 6-well plates in RPMI 1640 medium supplemented with 10% FBS without antibiotics overnight (day 0). On day 1, cells were transfected with 20 nM ASCT2 siRNA or negative control siRNA. On the second day, enzalutamide (20 μM) was added to each well. Cell proliferation was evaluated on day 4.

### 2.4. Western Blotting

To perform immunoblotting of enzalutamide-treated LNCaP lysates, cells (20 × 10^4^ per dish) were first seeded in a 100-mm dish and grown in RPMI 1640 medium supplemented with 10% FBS with antibiotics overnight (day 0). On day 1, 10 μM enzalutamide was added to each dish. Cells were harvested on day 3.

To perform immunoblotting of LNCaP and 22Rv1 lysates after ASCT2 siRNA transfection, cells (20 × 10^4^ per dish) were first seeded in a 100-mm dish and grown in RPMI 1640 medium supplemented with 10% FBS or CSS without antibiotics overnight (day 0). On day 3, those cells were harvested for ASCT2 siRNA transfection, and on day 4 for the combination therapy of ASCT2 siRNA transfection (20 nM) and enzalutamide (20 μM).

Cells were collected and lysed using RIPA buffer (25 mM Tris, 0.1 M NaCl, 1% Triton X-100, 0.5% deoxycholic acid, 0.1% SDS, pH 7.4). Protein expression levels were evaluated using Western blotting. Forty micrograms of the total protein from each sample were loaded onto NuPAGETM 4%–12% Bis-Tris Protein Gels (Invitrogen). The following primary antibodies were used: ARV7 (1:1000; RevMAb Biosciences, San Francisco, CA, USA), ASCT2 (1:1000; Cell Signaling Technologies, Danvers, MA, USA), AR (1:1000; Abcam, Cambridge, UK), and β-actin (1:1000; Abcam). LuminoGraph I (ATTO, Tokyo, Japan) was used for the captures and analyses of images. Western blot quantification was performed with CS Analyzer4 (ATTO, Tokyo, Japan).

### 2.5. Biostatistical Analyses

Publicly available transcriptome data for prostate adenocarcinoma and related clinical features were obtained from the cBioPortal database (www.cbioportal.org, accessed on 2 April 2013) [25]. We selected “Prostate Adenocarcinoma (MSKCC, Cancer Cell 2010)”, which was reported by Taylor et al. [26], and analyzed alterations in the ASCT2 gene.

### 2.6. Statistical Analysis

All values are presented as the mean ± SD. The Student’s *t*-test was used for the statistical comparisons of the results. Western blot analyses were performed at least twice, and all other in vitro experiments were repeated at least in triplicate.

## 3. Results

### 3.1. Association between ASCT2 Expression and Biochemical Recurrence-Free Survival in PCa Patients with a High Gleason Score

**Figure 1:**
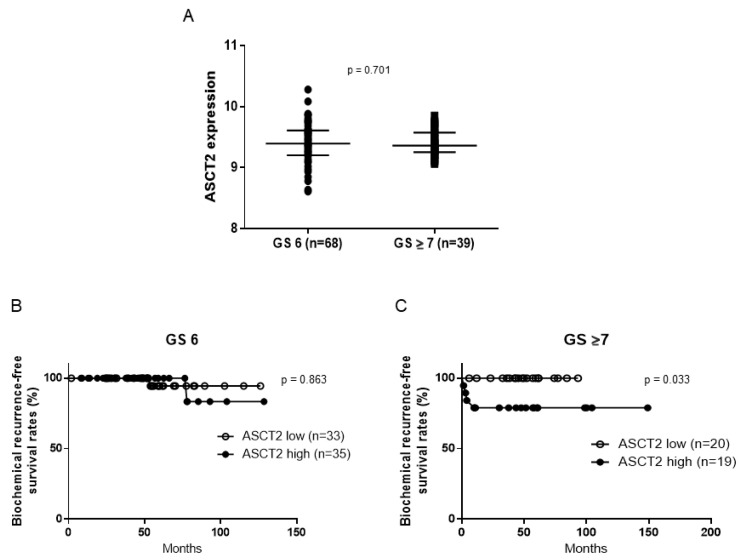
ASCT2 expression levels according to the cBioPortal database. (**A**) ASCT2 expression in the GS 6 group (*n* = 68) and GS ≥ 7 group (*n* = 39); (**B**) Kaplan–Meier curves for BCR-free survival according to ASCT2 expression level in the GS 6 group; (**C**) Kaplan–Meier curves for BCR-free survival according to ASCT2 expression level in the GS ≥ 7 group.

To assess the effect of ASCT2 expression levels on the oncological outcomes of PCa patients who underwent prostatectomy, we used the cBioPortal database. Of the 108 patients, the median ASCT2 expression level was 9.39 (8.61–10.28). Next, we evaluated the relationship between ASCT2 expression levels and Gleason score (GS). The median expression level of ASCT2 was 9.40 in the GS 6 group (*n* = 68) and 9.36 in the GS ≥ 7 group (*n* = 39), with no significant difference (*p* = 0.701) (Figure 1A). To assess whether ASCT2 expression affected biochemical recurrence (BCR)-free survival after prostatectomy, 108 patients were divided into two groups according to the median level of ASCT2 expression. Of the patients in the GS 6 category, no obvious difference was observed in BCR-free survival between the ASCT2-low (*n* = 33) and ASCT2-high groups (*n* = 35) (*p* = 0.863) (Figure 1B), although there was a significant difference between patients in the ASCT2-low (*n* = 20) and ASCT2-high groups (*n* = 19) in the GS ≥ 7 category (*p* = 0.033) (Figure 1C). Considering the results obtained from clinical data, we focused on ASCT2 and investigated its function in PCa cells in vitro.

### 3.2. ASCT2 Knockdown by siRNA in LNCaP and 22Rv1 Cells

**Figure 2:**
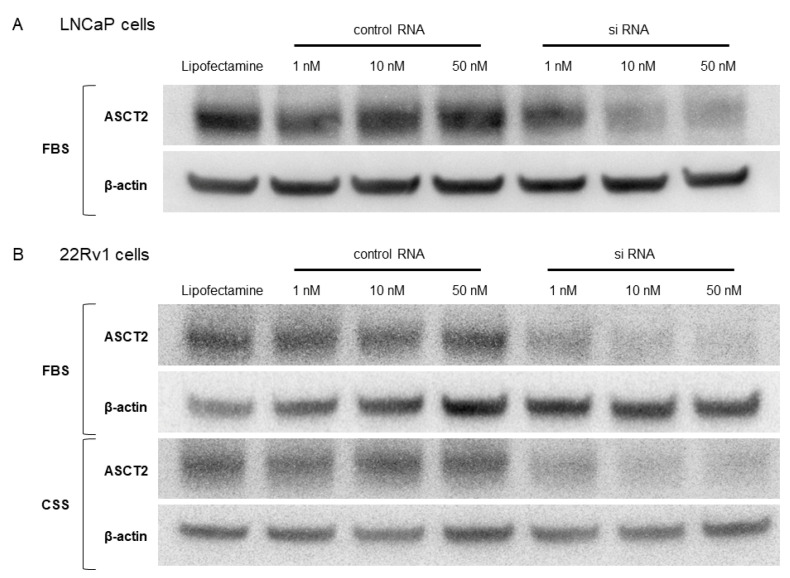
Knockdown of ASCT2 with siRNA transfection in LNCaP (**A**) and 22Rv1 (**B**) cells.

In order to investigate the biological role of ASCT2, chemically synthesized ASCT2 siRNA was transfected into LNCaP and 22Rv1 cells. Then, Western blotting was performed to examine the specificity and potency of ASCT2 siRNA in those cells. LNCaP cells in FBS medium and 22Rv1 cells in FBS or CSS medium were treated with ASCT2 siRNA or negative control siRNA at 1, 10, and 50 nM. The expression level of ASCT2 was suppressed in ASCT2 siRNA-transfected LNCaP cells in a dose-dependent manner compared to that in the negative control (Figure 2A). In 22Rv1 cells, the expression level of ASCT2 was stably suppressed at ASCT2 siRNA concentrations of 10 nM and 50 nM compared to that in the negative control cells in both FBS and CSS medium (Figure 2B).

### 3.3. Effects of ASCT2 Inhibition on LNCaP Cells

**Figure 3:**
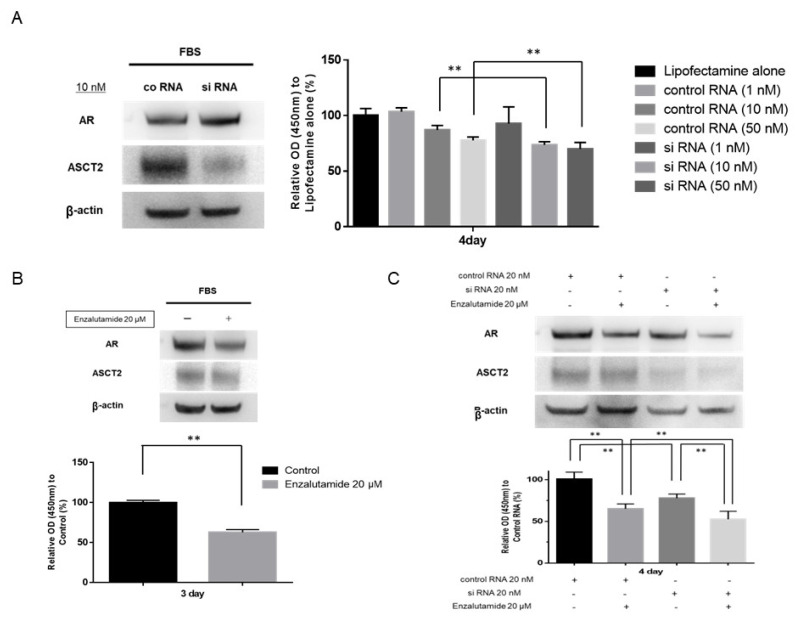
Effect of ASCT2 transfection and enzalutamide treatment in LNCaP cells. (**A**) Representative images of Western blot analyses of LNCaP cells after transfection of ASCT2 siRNA (10 nM) or negative control siRNA (10 nM). The proliferation of LNCaP cells transfected with ASCT2 siRNA (1, 10, 50 nM) or negative control siRNA (1, 10, 50 nM) or Lipofectamine alone was assessed on day 4 using the cell counting kit-8. The percentage of cells is expressed as proliferation activity relative to Lipofectamine alone. Histograms represent the mean ± SD (** *p* < 0.01). (**B**) Representative images of Western blot analyses of LNCaP cells after enzalutamide treatment (20 µM). AR, androgen receptor; FBS, fetal bovine serum. The proliferation of LNCaP cells after enzalutamide treatment (20 μM) or control (no treatment) was assessed on day 3 using the cell counting kit-8. The percentage of cells is expressed as proliferation activity relative to control. Histograms represent the mean ± SD (** *p* < 0.01). (**C**) Representative images of Western blot analyses of LNCaP cells after combination therapy with ASCT2 siRNA (20 nM) or negative control siRNA (20 nM) and enzalutamide treatment (20 μM).

Next, since ASCT2 expression was suppressed in ASCT2 siRNA-transfected LNCaP cells in a dose-dependent manner, we examined the effect of ASCT2 inhibition on LNCaP cells in FBS-containing medium. The expression levels of AR and ASCT2 were examined using Western blot analysis on day 3. Following inhibition of ASCT2 in LNCaP cells using 10 nM siRNA, ASCT2 expression stably decreased compared to that in cells treated with negative control siRNA, whereas AR expression was not affected after ASCT2 inhibition. Next, the growth ratio of LNCaP cells in FBS medium on day 4 after treatment with ASCT2 siRNA was compared to that of cells transfected with negative control siRNA. The growth of ASCT2 siRNA transfected LNCaP cells was significantly reduced compared to that of cells transfected with negative control siRNA, at both 10 nM and 50 nM (Figure 3A). These results indicated that ASCT2 inhibition alone might suppress the growth of LNCaP cells, partially via mechanisms independent of the AR signaling axis.

### 3.4. Effects of Enzalutamide on LNCaP Cells

Next, we evaluated the effect of enzalutamide on LNCaP cells in a medium containing FBS. The expression levels of AR and ASCT2 were examined using Western blot analysis on day 3. After treatment of LNCaP cells with enzalutamide (20 μM), the AR expression levels were stably suppressed, whereas the ASCT2 expression levels were not affected (Supplemental Figure S1). The LNCaP cell growth ratio in FBS medium on day 3 after the treatment with enzalutamide was then compared with that of untreated cells. The growth of LNCaP cells after enzalutamide treatment at concentrations of 20 μM was significantly reduced compared to untreated cells (*p* < 0.01) (Figure 3B).

### 3.5. Effect of Combination Therapy with ASCT2 siRNA and Enzalutamide on LNCaP Cells

We evaluated the effect of combination therapy with ASCT2 siRNA and enzalutamide on LNCaP cells in a medium containing FBS. One day after the treatment with ASCT2 siRNA or negative control siRNA, 20 μM enzalutamide was added to medium containing 10% FBS. Western blotting and cell growth assays were performed on days 3 and 4, respectively. As expected, the expression levels of AR and ASCT2 were stably suppressed by enzalutamide and ASCT2 siRNA, respectively. Moreover, ASCT2 expression levels were not affected after treatment with enzalutamide 20 μM (Supplemental Figure S1). Importantly, the AR expression level was significantly decreased compared to that after combination therapy with ASCT2 siRNA and enzalutamide. Cell growth on day 4 was significantly reduced after combination therapy with control RNA and enzalutamide or ASCT2 siRNA transfection alone as compared with control RNA transfection alone (both *p* < 0.01). The growth of LNCaP cells on day 4 following combination therapy with ASCT2 siRNA and enzalutamide treatment in FBS medium was significantly reduced compared to that following treatment with enzalutamide alone or ASCT2 siRNA transfection alone (*p* < 0.01, 0.01, respectively) (Figure 3C). These results suggested that the growth of LNCaP cells in FBS medium might be inhibited, partially because of a synergistic effect between ASCT2 inhibition and enzalutamide treatment.

The proliferation of LNCaP cells after combination therapy with ASCT2 siRNA (20 nM) or negative control siRNA (20 nM) and enzalutamide treatment (20 μM) or control (no treatment) was assessed on day 4 using the cell counting kit-8. The percentage of cells is expressed as proliferation activity relative to control. Histograms represent the mean ± SD (** *p* < 0.01).

### 3.6. Effects of ASCT2 Inhibition on 22Rv1 Cells

**Figure 4:**
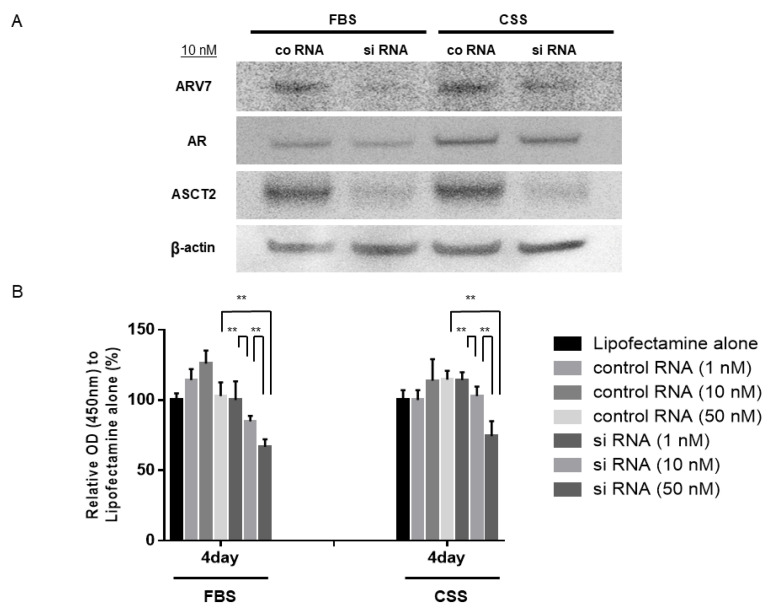
Effect of ASCT2 siRNA transfection in 22Rv1 cells. (**A**) representative images of Western blot analyses of 22Rv1 cells after transfection with ASCT2 siRNA (10 nM) or negative control siRNA (10 nM); (**B**) The proliferation of 22Rv1 cells transfected with ASCT2 siRNA (1, 10, 50 nM) or negative control siRNA (1, 10, 50 nM) or Lipofectamine alone was assessed on day 4 using the cell counting kit-8. The percentage of cells is expressed as proliferation activity relative to Lipofectamine alone. Histograms represent the mean ± SD (** *p* < 0.01).

Since the expression levels of ASCT2 were stably suppressed at ASCT2 siRNA concentrations of 10 nM and 50 nM compared to cells transfected with a negative control siRNA in both FBS and CSS medium, we examined the effect of ASCT2 inhibition on 22Rv1 cells in both media, to investigate the relationship between ASCT2 and ARV7. The expression levels of ARV7, AR, and ASCT2 were examined on day 3 using Western blot analysis. After inhibition of ASCT2 in 22Rv1 cells using 10 nM siRNA, the expression level of ASCT2 stably decreased compared to that following transfection with negative control siRNA in both FBS and CSS medium. In both media, ARV7 expression levels were significantly decreased after treatment with ASCT2 siRNA, whereas the expression levels of AR were not affected in response to ASCT2 inhibition (Figure 4A) (Supplemental Figure S1). Next, the 22Rv1 cell growth ratio in FBS and CSS medium on day 4 following treatment with ASCT2 siRNA was compared with that of cells transfected with negative control siRNA. In both media, the growth of 22Rv1 cells was reduced in a dose-dependent manner following treatment with ASCT2 siRNA. Moreover, following inhibition of ASCT2 by siRNA transfection, the growth of 22Rv1 cells was significantly suppressed compared cells transfected with negative control siRNA at a concentration of 50 nM, in both the FBS and CSS media (Figure 4B). These results indicate that ASCT2 inhibition suppressed cell growth not only in CSPC cells but also in ARV7-positive CRPC cells.

## 4. Discussion

In this study, we initially used the cBioPortal database to assess the effect of ASCT2 expression levels on the oncological outcomes of patients with PCa who underwent prostatectomy. Our analyses revealed a significant difference in BCR-free survival between patients in the ASCT2-low and ASCT2-high groups with GS ≥ 7. These results suggest that ASCT2 may affect PCa carcinogenesis, at least in the high GS category.

Next, we focused on ASCT2 expression and investigated its function in PCa cells in vitro. To investigate the biological role of ASCT2 in the CSPC stage, LNCaP cells were transfected with chemically synthesized ASCT2 siRNA and treated with enzalutamide. The present study demonstrated that ASCT2 inhibition suppresses the growth of CSPC cells. In LNCaP cells, growth after combination therapy with ASCT2 siRNA and enzalutamide was significantly reduced compared to that after treatment with enzalutamide alone or ASCT2 siRNA transfection alone. However, combination therapy with ASCT2 siRNA and enzalutamide might reduce LNCaP cell growth by approximately 50%. Considering this reduction in LNCaP cell growth, further studies, both in vitro and in vivo clinical studies, are required to evaluate this treatment.

Western blot analysis of LNCaP cells revealed that AR expression was not affected by ASCT2 inhibition and that ASCT2 inhibition alone might suppress the growth of LNCaP cells via mechanisms that are partially independent of the AR axis. Interestingly, however, the expression levels of AR were the lowest compared to the levels of the other proteins following combination therapy with ASCT2 siRNA and enzalutamide. Further experiments should be conducted to elucidate the exact relationship among ASCT2, AR, and AR antagonists; however, our present results suggest that the maximum reduction in the growth of LNCaP cell was obtained in response to the combination therapy with ASCT2 siRNA and enzalutamide, partially because of a synergistic effect between ASCT2 inhibition and enzalutamide treatment.

According to several recent reports, aerobic glycolysis, lipid metabolism, and other anabolic processes were stimulated by AR signaling [27,28,29]. Moreover, other reports demonstrated AR was working as a critical metabolic regulator by integrating metabolic profiling with genomic studies using LNCaP cells in order to identify transcriptional networks [28,30,31]. Massie et al. clarified a coordinated network of transcriptional changes conducted by AR, and it involved the upregulation of glucose, lipid, nucleotide, and amino acid metabolism, as well as cell cycle regulators [27]. Our present results demonstrating a possible synergistic effect between ASCT2 inhibition and enzalutamide treatment in LNCaP cells are consistent with these previous findings.

To investigate the biological role of ASCT2 in the CRPC stage, transfection of chemically synthesized ASCT2 siRNA was also performed in 22Rv1 cells, which are ARV7-positive. The present study demonstrated that ASCT2 inhibition suppressed the growth of 22Rv1 cells as well as LNCaP cells. Considering the results of our Western blot analyses that showed ARV7 expression levels to be significantly decreased after treatment with ASCT2 siRNA, suppressed growth of 22Rv1 cells may be induced by the reduction of ARV7 expression. Regarding the effect of ARV7 on 22Rv1 cells, we previously demonstrated that, after the combination therapy of AMACR inhibition and docetaxel treatment, the cell growth of 22Rv1 was significantly inhibited with decreased levels of ARV7 expression under androgen deprivation medium [32].

In the context of the contributions of AR and ARV7 to metabolism in PCa cells, our Western blot analysis showed that the expression levels of ARV7 significantly decreased after treatment with ASCT2 siRNA, whereas AR expression levels were not affected. Shafi et al. first showed their unique metabolic profiles and functions of AR and ARV7 [19]. They found that ARV7 and AR similarly stimulated not only growth and migration but glycolysis by measuring the extracellular acidification rate. However, increased dependence on glutaminolysis and reductive carboxylation to produce TCA metabolites were shown in ARV7 as compared with AR by their flux assays. Furthermore, decreased steady state citrate levels, despite an enhanced rate of its synthesis from glucose and glutamine, implies an increase in the utilization of this key TCA intermediate for generating biochemical components required for CRPC progression. Taking this into consideration, our result indicating that ASCT2 inhibition influenced expression levels of ARV7 but not AR in 22Rv1 cells could be reasonable. However, similarly to LNCaP cells, further research is needed in order to elucidate the relationship among ASCT2, AR, and ARV7, in 22Rv1 cells.

The present study is associated with several limitations that should be acknowledged. First, many confounding factors which may influence BCR should be appropriately controlled, in order to precisely assess the correlation between ASCT2 expression and clinical parameters. Second, the use of ASCT2 siRNA or enzalutamide were not evaluated enough, including their optimal concentration or infusion timings. Third, the relationship between ASCT2 and the AR axis or other AR-related signaling pathways in PCa cells has not been fully elucidated. Thus, further in vitro and in vivo studies are required to confirm the findings of this study.

## 5. Conclusions

In conclusion, our in vitro experiments demonstrated that ASCT2 inhibition significantly reduced the proliferation of both CSPC and CRPC cells. Furthermore, combination therapy with ASCT2 inhibition and enzalutamide treatment induced the maximum reduction in CSPC cell growth. Although further studies are needed, ASCT2 could be a useful target for both CSPC and CRPC patients.

## Data Availability

No new data were created or analyzed in this study. Data sharing is not applicable to this article.

## Supplementary Materials

The following supporting information can be downloaded at: https://www.mdpi.com/article/10.3390/jcm11185466/s1, Figure S1: Western blot quantification.
