# Supplementary material for: Alanine-Serine-Cysteine Transporter 2 Inhibition Suppresses Prostate Cancer Cell Growth In Vitro
Source: J Clin Med. 2022 Sep 16;11(18):5466. doi: 10.3390/jcm11185466 (PMC9501406; doi:10.3390/jcm11185466)
Supplement: Supplementary file 1 [file jcm-11-05466-s001.zip › jcm-1888560-supplementary.pdf]

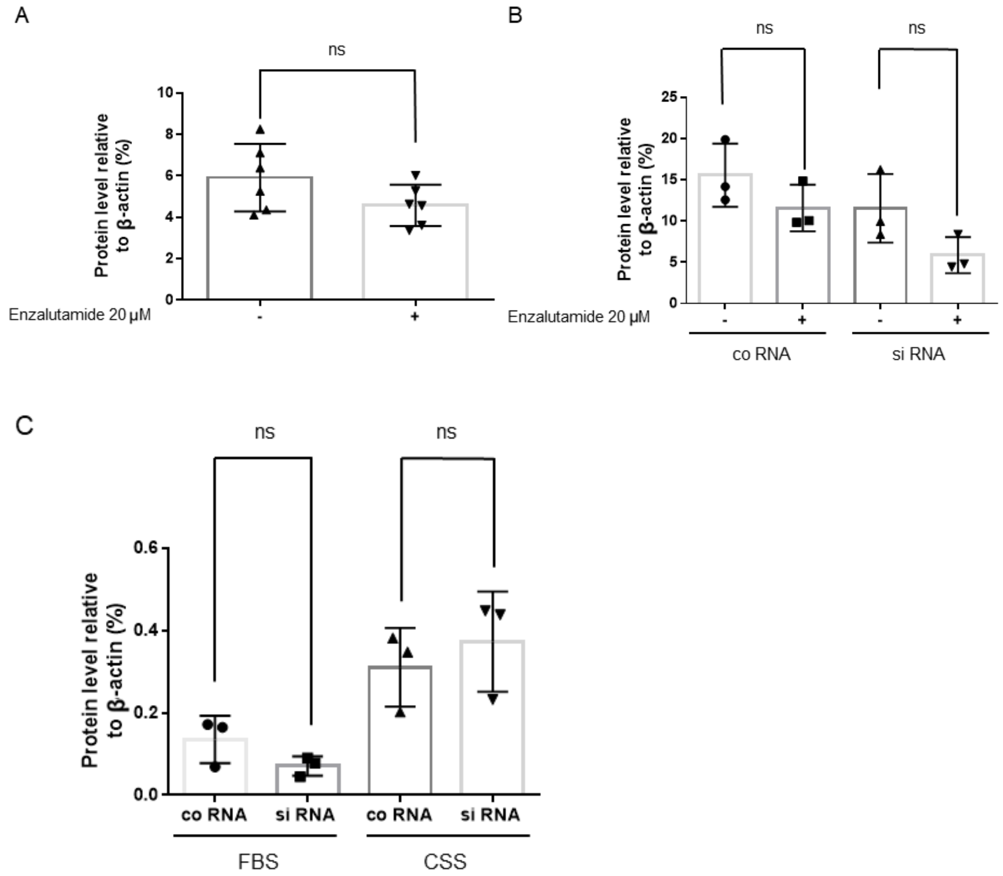

Supplemental Figure S1. Western blot quantification. (**A**) ASCT2 expression levels relative to β-actin were measured with or without enzalutamide 20 μM in LNCaP cells. Histograms represent the mean ± SD (NS: no significant difference). (**B**) ASCT2 expression levels relative to β-actin were measured with or without enzalutamide 20 μM, after treatment with ASCT2 or negative control siRNA in LNCaP cells. Histograms represent the mean ± SD (NS: no significant difference). (**C**) AR expression levels relative to β-actin were measured after treatment with ASCT2 or negative control siRNA in FBS or CSS media, in 22Rv1 cells. Histograms represent the mean ± SD (NS: no significant difference).
